# How ICU Patient Severity Affects Communicative Interactions Between Healthcare Professionals: A Study Utilizing Wearable Sociometric Badges

**DOI:** 10.3389/fmed.2020.606987

**Published:** 2020-12-03

**Authors:** Eiji Kawamoto, Asami Ito-Masui, Ryo Esumi, Hiroshi Imai, Motomu Shimaoka

**Affiliations:** ^1^Departments of Molecular and Pathobiology and Cell Adhesion Biology, Mie University Graduate School of Medicine, Tsu, Japan; ^2^Departments of Emergency and Disaster Medicine, Mie University Graduate School of Medicine, Tsu, Japan

**Keywords:** wearable, inter-professional collaboration, communication, human behavior, critical care

## Abstract

Numerous factors affecting the interactions between healthcare professionals in the workplace demand a comprehensive understanding if the quality of patient healthcare is to be improved. Our previous cross-sectional analysis showed that patient severity scores [i.e., Acute Physiology and Chronic Health Evaluation (APACHE) II] in the 24 h following admission positively correlated with the length of the face-to-face interactions among ICU healthcare professionals. The present study aims to address how the relationships between patient severity and interaction lengths can change over a period of time during both admission and treatment in the ICU. We retrospectively analyzed data prospectively collected between 19 February to 17 March 2016 from an open ICU in a University Hospital in Japan. We used wearable sensors to collect a spatiotemporal distribution dataset documenting the face-to-face interactions between ICU healthcare professionals, which involved 76 ICU staff members, each of whom worked for 160 h, on average, during the 4-week period of data collection. We studied the longitudinal relationships among these interactions, which occurred at the patient bedside, vis-à-vis the severity of the patient's condition [i.e., the Sequential Organ Failure Assessment (SOFA) score] assessed every 24 h. On Day 1, during which a total of 117 patients stayed in the ICU, we found statistically significant positive associations between the interaction lengths and their SOFA scores, as shown by the Spearman's correlation coefficient value (R) of 0.447 (*p* < 0.01). During the course of our observation from Day 1 to Day 10, the number of patients (*N*) who stayed in the ICU gradually decreased (*N* = 117, Day1; *N* = 10, Day 10), as they either were discharged or died. The statistically significant positive associations of the interaction lengths with the SOFA scores disappeared from Days 2 to 6, but re-emerged on Day 7 (*R* = 0.620, *p* < 0.05) and Day 8 (*R* = 0.625, *p* < 0.05), then disappearing again on Days 9 and 10. Whereas all 6 SOFA sub-scores correlated well with the interaction lengths on Day 1, only a few of the sub-scores (coagulation, cardiovascular, and central nervous system scores) did so; specifically, those on Days 7 and 8. The results suggest that patient severity may play an important role in affecting the interactions between ICU healthcare professionals in a time-related manner on ICU Day 1 and on Days 7/8.

## Introduction

Facilitating positive and productive interactions between healthcare professionals in the workplace constitutes a cornerstone of successful inter- and intra-professional collaborations, and thereby, of improving the quality of patient healthcare (1). A better understanding of the underlying mechanisms by which clinician interactions in the workplace are facilitated is essential for filling critical knowledge gaps that might hamper effective policy planning to best realize a productive work environment (2, 3). However, an objective assessment of the factors affecting these interactions has remained technically challenging.

The study of human interactions in small collocated face-to-face groups has been recognized for 40 years as a firm basis for understanding collaborative organizational behavior (4). However, only recently has a comprehensive analysis of face-to-face interactions in the workplace been made possible thanks to emerging wearable sociometric badge technologies (5–9). Sociometric badge-based analyses suggest that cohesive face-to-face interactions enable information-rich communication and knowledge transmission, thereby aiding the resolution of complex problems (10) such as those found in ICUs. We have recently demonstrated the feasibility of sociometric badges for objectively assessing interactions between ICU healthcare professionals (9). Importantly, the length of the face-to-face interactions between ICU staff and a given patient during the first 24 h after admission has been shown to positively correlate with that patient's Acute Physiology and Chronic Health Evaluation II (APACHE II) score (9).

Capitalizing on our previous study (9), which focused on a cross-sectional analysis of patient severity (i.e., APACHEII) vs. face-to-face interactive relationships on Day 1, here we performed a longitudinal analysis to investigate the relationship between patient severity [i.e., the sequential organ failure assessment (SOFA) score] and face-to-face interactions during the first 10 days following ICU admission. Interestingly, our longitudinal analysis revealed a biphasic pattern (Day 1 and Days 7/8) of positive associations between patient severity and face-to-face healthcare professional interactions in the ICU.

## Materials and Methods

### Participants

This study was approved by the Mie University Human Research Ethics Committee (approval no. 2978) as previously described (9). Briefly, the investigation was conducted within the premises of an ICU at Mie University Hospital, a tertiary emergency care department in Japan. This department functions as a referral center for critical patients with conditions such as high-degree burns, post-cardiac arrest, septic shock, and life-threatening trauma. All staff members working within the ICU (physicians, nurses, nursing assistants, pharmacists, medical engineers, desk clerks, and secretaries) were included in the study. Written consent was obtained from all participants. A few staff who did not agree to participate wore mock badges with no sensor functions in order to maintain confidentiality. A total of 76 medical professionals participated in the research project: 15 attending physicians, 39 nurses, 4 senior residents, 1 resident, 4 nursing assistants, 8 medical engineers, 2 receptionists, 1 pharmacist, and 2 secretaries (Table 1). All participants wore the badges on the front of their clothing for 4 weeks during their working hours, including breaks.

**Table 1:** Demographic characteristics of participants.

**Healthcare professionals (experience)**	***N* (%)**	**Face-to-face interaction lengths** **(mins/person/day, mean)**
Attending physicians	15	
5–15 years	9 (60)	33.4
>15 years	6 (40)	41
Senior residents	4	
<5 years	4 (100)	54.6
Resident	1	
<5 years	1 (100)	84.4
Nurses	39	
<5 years	9 (23)	133.4
5–15 years	26 (67)	139.3
>15 years	4 (10)	193.9
Nursing assistants	4	
<5 years	4 (100)	163
Medical engineers	8	
<5 years	4 (50)	42.4
5–15 years	2 (25)	13.2
>15 years	2 (25)	48.1
Receptionists	2	
5–15 years	2 (100)	71.7
Pharmacist	1	
5–15 years	1 (100)	22.3
Secretaries	2	
5–15 years	2 (100)	23.5

### Wearable Sensors and Data Acquisition

Wearable sociometric sensor badges (Business Microscope™, Hitachi, Ltd., Tokyo) were used as previously described (9, 11–13). The badges enable the continuous monitoring of interactions between two or more interfaces and provide information on face-to-face interactions. They were previously described in investigations that sought to quantitatively measure face-to-face interactions and communication within our group (9, 11–13) and by others (14). The badges were attached to the participants' front pockets so that they would not physically hinder any occupational ICU duties. The badges do not interfere with medical devices; hence, these badges enable the safe collection of communication and behavioral data in a real-life ICU setting. The wearer's physical movements are captured via a three-axis micro electro-mechanical acceleration sensor, which is used to detect individual activities. Six infrared data association transceivers on the front of the badge face at different angles and are used to detect interpersonal interactions. Four infrared transceivers on the front of the beacon, facing at slightly different angles, create a detection range encompassing 60 degrees horizontally and 30 degrees vertically. Data on who met whom, when, and for how long can be collected. Location information is obtained by using infrared beacons set at particular locations within the ICU. A total of 249 infrared beacons were widely placed throughout the ICU in locations such as the patients' bedsides, the staff room, the conference room, and all other areas used daily by employees. As the infrared beam signals transmit up to 2 m, and cannot travel through any solid objects, multiple beacons were placed in each area to cover any interactions. As beacons between the nurses' station, bedsides, and other areas were distant enough (e.g., >4 m), any overlapping detections were excluded. Separation walls between each bed completely obstructed the signals from neighboring beds, thereby allowing us to measure the lengths of the specific interactions that occurred with a given patient for any relevant duration. With the use of this badge and the infrared beacons, the researchers were able to measure the duration and location of all face-to-face communications among the study's participants.

The badges capture the physical movements of ICU staff using built-in acceleration sensors. The zero-crossing count, defined as the number of times the acceleration signal crossed the zero-level per unit time, was employed to determine activity levels: the higher the count, the more active the person's bodily movements. Each staff member's activity level was evaluated on a minute-to-minute base and classified into one of two states in accordance with the zero-crossing count: active or non-active. The threshold frequency for the count was set to 2 Hz on the basis of the results obtained from previous studies (11, 13), which demonstrated this to be the level at which active motion, such as conversation accompanied by gestures, could be distinguished from quieter motions such as keyboard typing. Therefore, an activity level with a zero-crossing frequency >2 Hz for 1 min was judged to be in the active state, and otherwise was regarded as being in the inactive state. Detection was fixed for conversations longer than 1 min since a threshold value set to 1 min or less might have falsely recorded a simple instance of staff members passing each other in the ICU as active face-to-face communication. The captured data were stored in built-in 32-MB flash memory cards and were offloaded for database transfer when the badges were placed in the charging cradle overnight.

The face-to-face interactions were captured using the infrared data association transceivers embedded in the badges. Three measures were used to evaluate the cohesiveness or liveliness of these mutual face-to-face interactions: degree, clustering coefficient, and geodesic distance (15). Two people were considered to have “actively communicated” with each other if there was a face-to-face event between them that exceeded a predefined threshold (i.e., 2 Hz) of more than 1 min. Active face-to-face interactions were previously typified by gesture-aided conversation (9, 11–13).

### Data Analysis

It is generally assumed that severely ill patients demand elevated levels of monitoring and medical attention; however, it remains to be elucidated how the interactions between ICU staff might be affected by the severity of the patient whose care they are charged with during ICU admission. Our previous work showed a positive association between the interaction length and the APACHE II score on Day 1; however, how the association would unfold during the course of ICU admission had yet to be shown. To address this question, we extracted data on the active interactions that occurred at the bedside of each ICU patient (Table 2) on each day up to Day 10 following ICU admission. We then studied the correlation between the interaction levels (a daily sum of the active face-to-face interactions that occurred at a given patient's bedside on a specific ICU day) and the severity of the patient's condition on a specific ICU day using the sequential organ failure assessment (SOFA) score for that patient.

**Table 2:** Demographic characteristics of patients.

	**All**	**SOFA < 8**	**SOFA ≧ 8**
Patients, *n*	117	85	32
Age(median), years	67	66	71
Gender, male/female	74/43	57/28	17/15
**Reasons for ICU admission**			
Medical	51	36	15
• Acute respiratory failure	9	5	4
• Shock	30	19	11
• Coma	16	12	4
• Acute kidney injury	1	1	0
Surgical	53	43	10
Trauma	9	6	3
ICU days (average)	3.2	2.3	5.5
Deaths	6	0	6
**The number of patients requiring invasive treatments at ICU**			
• Mechanical ventilation	52	26	26
• Central venous catheter	80	48	32
• Continuous hemodiafiltration	11	5	6
Face-to-face interaction lengths (mins/patient/day, median)	53	42	77

Patient severity data (SOFA score) (16) were extracted from electronic ICU records. Missing data in the records were handled as previously described (17). A large dataset involving 76 ICU staff members, each of whom worked for 160 hours on average during the 4-week period of data collection, was compiled, resulting in a total of 729,600 min · persons (min · person). This corresponds to more than 400 million data points, as has been previously described (9). These data points were re-analyzed here to study the longitudinal association of patient severity with face-to-face interactions between ICU healthcare professionals.

SPSS software v.25.0 (SPSS, Inc., Chicago, IL, USA) was used to perform statistical analyses. Spearman's correlation coefficients were calculated to evaluate the correlations. The Wilcoxon and Mann-Whitney tests were employed for within-group comparisons. A probability (*p*) value of <0.05 was considered statistically significant.

## Results

**Figure 1:**
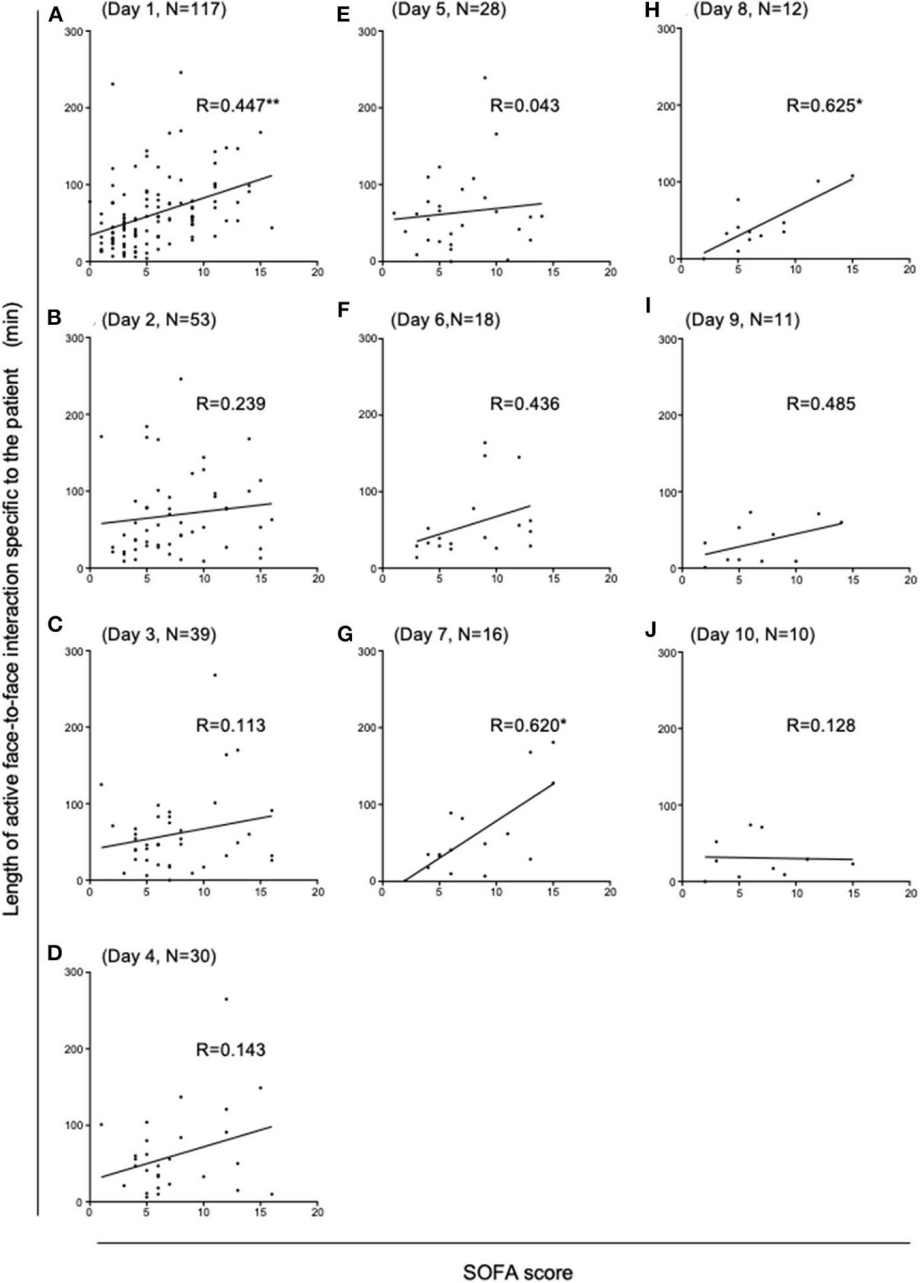
Correlation analysis between the SOFA scores and the lengths of active face-to-face interactions during ICU admission. The panel of scatter plots with a linear regression line illustrates how the lengths of face-to-face active interactions that occurred at each patient bedside during ICU Days 1 through 10 **(A–J)** correlated with that patient's SOFA score. Statistically significant positive correlations were observed on ICU Day 1 **(A)**, Day 7 **(G)** and Day 8 **(H)** (*p < 0.05 and **p < 0.01). Spearman's correlation coefficient values (*R*) and the number of patients (*N*) are shown for each ICU Day.

**Figure 2:**
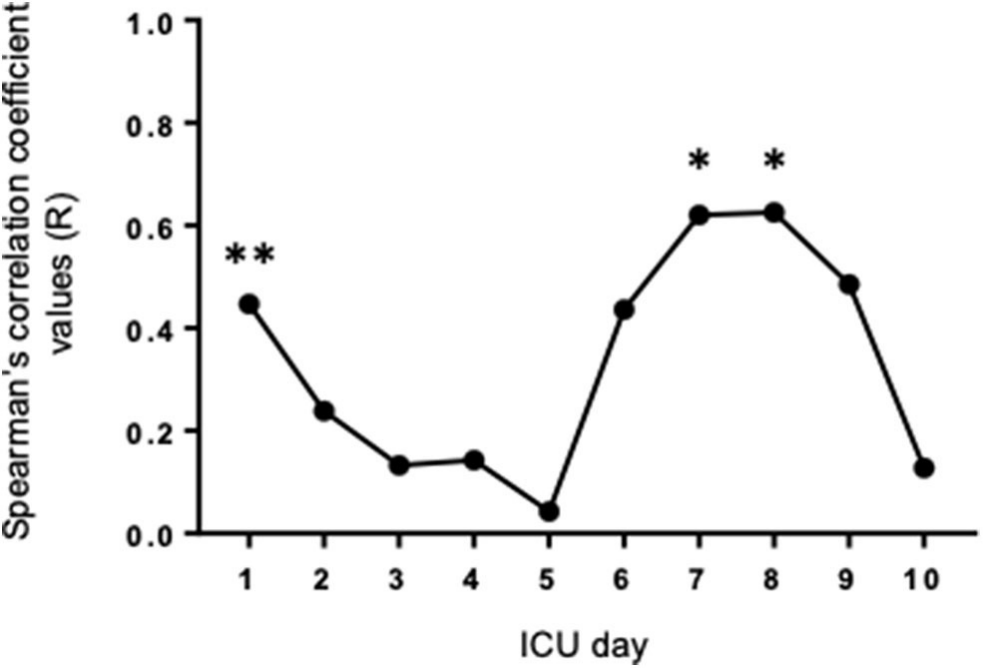
Strengths of associations between the SOFA scores and the face-to-face interactions changing biphasically during ICU Days 1 through 10, as represented by the daily changes of the Spearman's correlation coefficient values (*R*). Statistically significant positive correlations were observed on Days 1, 7, and 8 (*p < 0.05 and **p < 0.01).

Active face-to-face interactions were recorded mostly at the central nurses' station (43.4%), followed by patient bedsides (35.9%), the reception area (5.8%), the nurses' lounge (4.5%), examination-and-procedure rooms (4.5%), and the conference room (2.3%), as shown in our previous report (9). By concentrating on the interactions at patient bedsides, we have studied how the interaction lengths correlated with the patient severity assessed by the SOFA score, thereby following up on and extending our previous report on the APACHE II score. In contrast to the APACHE II score, which by definition is calculated only at 24 h following admission, the SOFA scores can be calculated every 24 h until discharge (17) (Table 3), thereby enabling us to carry out a longitudinal analysis into the relationship between patient severity and interaction lengths. Consistent with our previous report on the APACHE II score, we found a significant positive correlation between active interaction levels and the SOFA score on Day 1 (Spearman correlation *R* = 0.447, *P* < 0.01) (Figure 1). Thereafter, however, on Days 2 through 6 significant correlations between the SOFA scores and the interaction lengths disappeared (Figure 1). Subsequently, the positive correlations re-appeared on Days 7 and 8, and then disappeared on Days 9 and 10 (Figure 1). It is worth noting that, despite a progressive decrease in the number of patients who remained under treatment in the ICU (Figure 1) during Days 1 through 10, the strengths of the associations increased to statistically significant levels in a biphasic manner on Day 1 and Days 7/8 (Figure 2).

**Table 3:** Median SOFA scores from day 1 to 10.

**ICU day**	**1**	**2**	**3**	**4**	**5**	**6**	**7**	**8**	**9**	**10**
SOFA scores (median)	5	7	7	6	6	9	7	6	6	7

We have carried out a sub-group analysis that concentrated only on patients who stayed in the ICU for 7 days or longer (Table 4). In those patients we found that the significant correlation between patient severity and face-to-face interactions did not become apparent until Day 7 (Table 5). No significant associations were observed on Days 1 through 6, thus suggesting that the biphasic emergence of the significant associations observed across the entire patient populations is not attributable to merely a specific group of patients such as those who remained in the ICU for longer periods.

**Table 4:** Demographic characteristics of a sub-group of 16 patients who stayed in the ICU for 7 or more days.

	**All**	**SOFA < 8**	**SOFA ≧ 8**
Patients, *n*	16	9	7
Age (median), years	70	66	75
Gender, male/female	12/4	7/2	5/2
**Reason for ICU admission**			
Medical	9	4	5
• Acute respiratory failure	1	0	1
• Shock	6	2	4
• Coma	4	4	0
• Acute kidney injury	0	0	0
Surgical	3	2	1
Trauma	2	1	1
ICU days (average)	11.3	9.6	13.6
Deaths	1	0	1
**The number of patients requiring invasive treatments at ICU**			
• Mechanical ventilation	13	7	6
• Central venous catheter	14	8	6
• Continuous hemodiafiltration	3	0	3
Face-to-face interaction lengths (mins/patient/day, median)	38	35	62

**Table 5:** A sub-group correlation analysis focusing on those 16 patients who were admitted to the ICU for 7 or more days.

**ICU Day**	**1**	**2**	**3**	**4**	**5**	**6**	**7**
R	0.234	−0.022	0.202	0.239	−0.015	0.434	0.620^*^

R, Spearman's correlation coefficient;

* *p < 0.05*.

**Figure 3:**
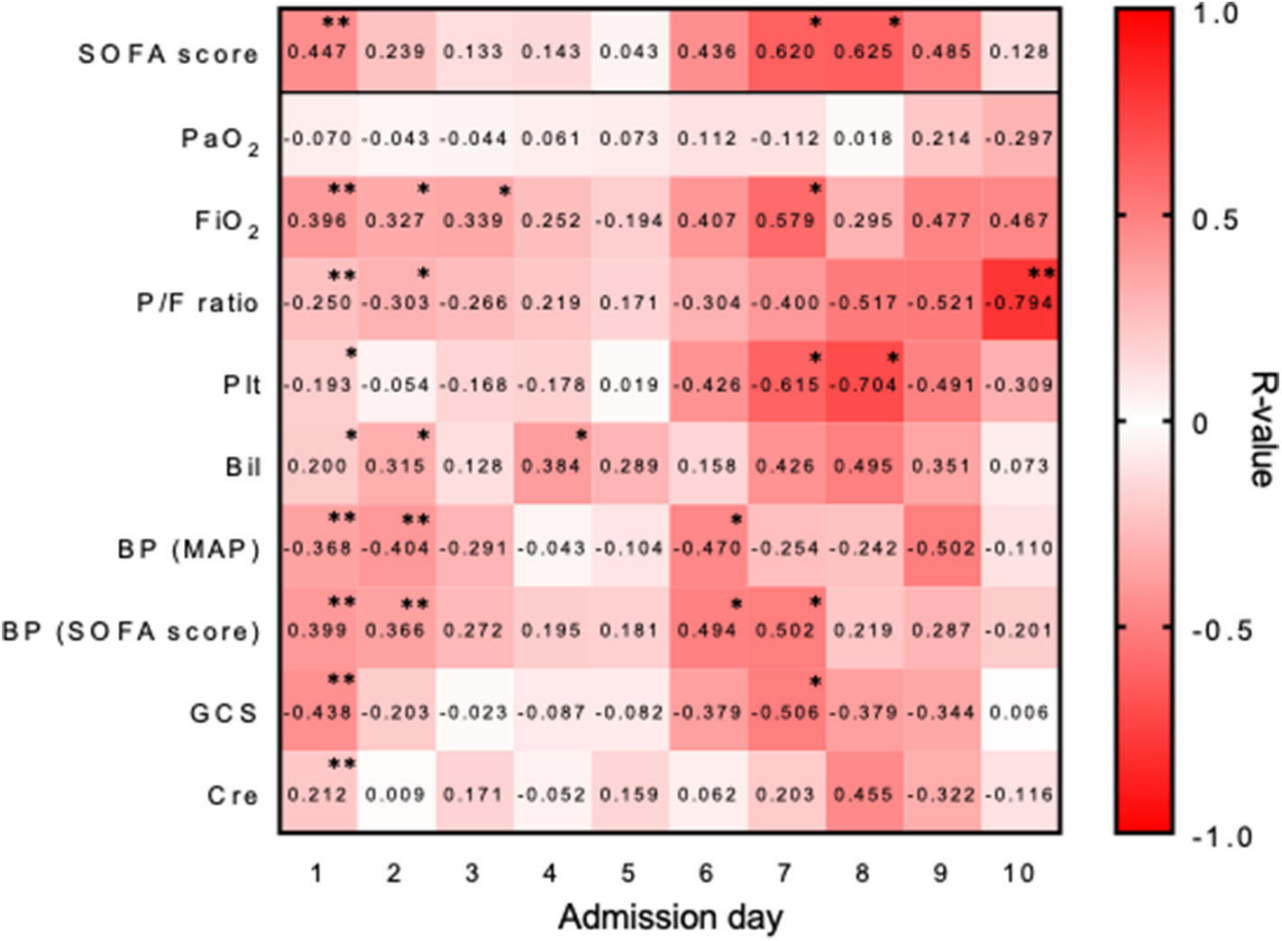
Correlations of the SOFA sub-scores with the face-to-face interactions. A heatmap representation showing the strengths of associations between the SOFA sub-scores and the interactions, as represented by the Spearman's correlation coefficient values (*R*). Statistically significant correlations are marked with asterisks (*p < 0.05 and **p < 0.01). PaO_2_, partial pressure of arterial oxygen; FiO_2_, fraction of inspired oxygen; P/F ratio, the ratio of arterial oxygen partial pressure to fractional inspired oxygen; Plt, blood platelet count; Bil, blood bilirubin concentration; BP (MAP), blood pressure (mean arterial pressure); BP (SOFA score), type(s) of vasopressor(s) administered; GCS, Glasgow coma scale; Cre, blood creatinine concentration.

The SOFA total scores were determined based on the sum of 6 different sub-scores: each one is uniquely designed for evaluating the organ functions of the respiratory (PaO_2_/FiO_2_), central nervous (Glasgow coma scale), cardiovascular (mean arterial pressure or vasopressors required), liver (serum bilirubin), coagulation (blood platelet counts), and kidney (serum creatinine) systems. To better understand how the associations between the different organ systems and the face-to-face interactions, we studied the correlation between each organ system sub-score and the corresponding face-to-face interactions (Figure 3). For example, on Day 1 not only did the SOFA total scores strongly correlate with the face-to-face interactions, but all 6 sub-scores did so as well. On Days 2 through 6, although the SOFA total scores did not strongly correlate with the interactions, the respiratory system sub-score did so on Day 2; those of the cardiovascular system did so on Days 2 and 6; and those of the liver system did so on Days 2 and 4. On Days 7 and 8, during which the SOFA total scores strongly correlated with the face-to-face interactions, thereby revealing a second peak (Figure 3), the sub-scores of the coagulation systems failed to do so on either day, while the cardiovascular and central nervous systems did so on Day 7. Thus, whereas the early correlation on Day 1 involved dysfunctions across all six organ systems, the late correlations observed on Days 7/8 involved dysfunctions of only select organ systems such as the coagulation, cardiovascular, and central nervous systems.

## Discussion

### Sociometric Badges to Study Interactions Among Healthcare Professionals

Inter- and intra-professional communication and interactions play prominent roles in promoting not only the transfer and exchanges of clinical information, but also the multi-disciplinary collaborations carried out in ICUs. Communication and collaboration between medical staff have been previously examined manually by questionnaires and/or observations (1, 18), making it difficult to perform objective and comprehensive/continuous measurements of communication between medical staff. Applications of novel technologies to address this problem could better our understanding of communication and interactions in the workplace, thereby improving the quality of research on the impact of practice-based interventions on collaborative behaviors. The wearable sociometric sensor badges used in the current study represent such an emerging technology, as they are designed to monitor interactions objectively and comprehensively. Their main innovation involves an acceleration detector capable of measuring the intensities of bodily motions associated with verbal communication. In this way, they can serve as a reliable surrogate measure of active communication. Our previous studies examined the intensities of bodily motions associated with a range of human behaviors from sleeping, listening, talking with and without dynamic gesture, rushed walking, to running (11, 13). The study established the threshold of 2 Hz to reliably distinguish talking with dynamic gestures (i.e., active communication) from talking without dynamic gestures or just listening (11, 13). By combining this data with information from the infrared sensors used to detect face-to-face interactions, these wearable sociometric sensor badges enabled very specific measuring of active face-to-face interactions, while ruling out such artifacts as irrelevant bodily motions such as rushed walking and running (11).

### Implication of the Positive Association Between the Patient Severity and the Interaction Lenghts Among ICU Healthcare Professionals

By using the sociometric wearable sensors worn by ICU healthcare professionals, the present study has shown that the significant positive associations between SOFA scores and face-to-face interactions were evident not only on Day 1, but also on Days 7/8. Interactions between ICU staff at patient bedsides during formal and informal clinical rounds are important for transferring critical information to manage patients. The more severe a patient's condition, the more communication between ICU staff is required to transmit patient information and discuss the management and care of said patient, which might explain the positive associations observed between the SOFA scores and the interaction lengths. Our finding that the strengths of the associations between severity and face-to-face interactions changed periodically, at an interval of a week, is novel and of great interest. After the first positive association was observed on Day 1, the second positive association was observed at an interval of approximately 1 week, on Days 7/8. A plausible explanation for a biphasicity occurring at weekly intervals might be that whereas patient information is transferred during daily rounds, extensive discussions that review and update patient history and clinical problems tend to be carried out weekly in some ICUs (19, 20) including our own. Such weekly discussions about patients admitted for longer time periods can occur either formally or informally. For example, the biphasic changes may be consistent with the following scenario that exemplifies what could happen in the ICU: on Day 1, severely ill patients warrant intensive discussion to exchange a considerable amount of information to implement an initial treatment plan; on Days 2 to 6, ICU staff tend to follow this initial plan, usually requiring less discussion as only minor modifications of the plan are typically required; and on Days 7 and 8, a weekly extensive assessment takes place regarding how well the initial plan is succeeding, possibly involving discussions about any needed modifications for those patients who remain severely ill. In this way, an understanding of the patient factors affecting information transfer is of great clinical importance. Patient severity, as represented by the SOFA score, is considered an important factor demanding increased levels of information transfer in an ICU setting. Increased patient severity demands more prolonged communication between ICU staff, which may increase staff workload and stress. For example, a previous report found that uncertainty and lack of control are among the major elements contributing to stressful work environments in ICUs (21). Indeed, as SOFA ≧ 8 predicts mortality rates > 20%, thus signifying a group of more severely ill patients (22), the interaction lengths between ICU healthcare professionals proved significantly longer than those with SOFA < 8 (Table 2). Therefore, our results may help to identify/target those specific situations and time points in the ICU work environment that would benefit most from interventions aimed at improving information transfer in hospitals (2, 23–25). As any intervention carries with cost, a better understanding of the patient factors affecting information transfer between healthcare professionals will help optimize strategies for applying such interventions in clinical settings.

### Limitations of the Present Study

There are a few limitations in the present study. Despite the increased utility of various sociometric sensors for studying human behaviors and communication (11–13, 26, 27), the face-to-face active interactions measured by these wearable badges may not perfectly mirror the communication and interactions assessed by manual methods such as observations and surveys. Active listening (28) and body language (29) might not be recorded as active interactions. The wearables did not include digital online information exchange or communication through emails, instant messaging, and microblogging, which have rapidly become dominant tools in modern societies. In addition, the wearable sensors record only meta data associated with communication regardless of the actual content (e.g., negative vs. positive sentiments, etc.). Nevertheless, face-to-face interactions influence people and remain a crucial means of transmitting valuable information. Indeed, face-to-face interactions are critical to the development of trust in organizations (30), as some of the most important human interactions in the workplace still take place offline. In fact, the majority of inter-professional communications conducted in ICUs are currently done offline. Another limitation to the present study may lie in the SOFA scoring system. Although SOFA scores have been shown to correlate with patient outcomes (16), whether the lengths of the interactions between healthcare professionals correlate with said outcomes remains to be elucidated. In addition, the SOFA scoring system itself may be prone to reliability issues such as inter-examiner variations (31). Future prospective investigations involving multiple ICUs across different countries are needed to confirm and generalize the results of our study (e.g., the biphasic/cyclic emergence of positive associations between patient severity and face-to-face interactions). A better understanding of the spatiotemporal distributions of the positive correlations between SOFA scores and the intensities of communication by medical staff could help optimize ICU resource allocation and policies (i.e., staffing, team member composition, and patient assignment) in order to better match patient severity and ICU workloads.

## Conclusions

Patient severity may play an important role in affecting the interactions between ICU healthcare professionals in a time-related manner on ICU Day 1 and on Days 7/8. Indeed, it constitutes an important factor to address if effective policy planning productive work environment are to be successfully realized.

## Data Availability Statement

The raw data supporting the conclusions of this article will be made available by the authors, without undue reservation.

## Ethics Statement

The studies involving human participants were reviewed and approved by Mie University Human Research Ethics Committee (approval no. 2978). The patients/participants provided their written informed consent to participate in this study.

## Author Contributions

EK, AI-M, and MS designed the study. EK, AI-M, RE, and HI collected the data. EK and MS analyzed the data. EK and MS wrote the manuscript. EK, AI-M, RE, HI, and MS obtained funding. All authors contributed to the article and approved the submitted version.

## Conflict of Interest

The authors declare that the research was conducted in the absence of any commercial or financial relationships that could be construed as a potential conflict of interest.
